# Absorption, distribution, metabolism and excretion of peginesatide, a novel erythropoiesis-stimulating agent, in rats

**DOI:** 10.3109/00498254.2011.649310

**Published:** 2011-12-22

**Authors:** Kathryn W. Woodburn, Christopher P. Holmes, Susan D. Wilson, Kei-Lai Fong, Randall J. Press, Yuu Moriya, Yoshihiko Tagawa

**Affiliations:** 1 Affymax Inc., Palo Alto, CA, USA; 2 Aclairo Pharmaceutical Development Group, Inc., Vienna, VA, USA; 3 Accellient Partners LLC, Waltham, MA, USA; 4 Aptuit, Kansas City, MO, USA; 5 Takeda Pharmaceutical Company Ltd, Osaka, Japan

**Keywords:** Erythropoiesis, biodistribution, quantitative whole-body autoradiography, bone marrow

## Abstract

1. The pharmacokinetics(PK) (absorption, distribution, metabolism, excretion) of peginesatide.a synthetic, PEGylated, investigational, peptide-based erythropoiesis-stimulating agent (ESA), was evaluated in rats. The PK profile was evaluated at 0.1-5 mg·kg^−1^ IV using unlabeled or [^14^C]-labeled peginesatide. Mass balance, tissue distribution and metabolism were evaluated following IV administration of 5 mg·kg^−1^ [^14^C]-peginesatide, with tissue distribution also evaluated by quantitative whole-body autoradiography (QWBA) following an IV dose of 17 mg·kg^−1^[^14^C]-peginesatide.
2. Plasma clearance was slow and elimination was biphasic with unchanged peginesatide representing >90% of the total radioactivity of the total radioactive exposure. Slow uptake of the radiolabeled compound from the vascular compartment into the tissues was observed.
3. Biodistribution to bone marrow and extramedullary hematopoietic sites, and to highly vascularized lymphatic and excretory tissues occurred.
4. A predominant degradation event to occur *in vivo* was the loss of one PEG chain from the branched PEG moiety to generate mono-PEG.
5. Renal excretion was the primary mechanism (41%) of elimination, with parent molecule (67%) the major moiety excreted.
6. In conclusion, elimination of [^14^C]-peginesatide-derived radioactivity was extended, retention preferentially occurred at sites of erythropoiesis (bone marrow), and urinary excretion was the primary elimination route.

## Introduction

Erythropoietin (EPO) is a glycoprotein hormone synthesized by the kidney in response to hypoxia that regulates the proliferation, differentiation, and survival of eryth-roid progenitor cells (Krantz 1991). The etiopathology of chronic kidney disease (CKD)-associated anemia is related to inadequate generation of erythropoietin by the diseased kidney. Anemia impairs cognitive function and is associated with an increased incidence of cardiovascular complications (Locatelli et al. 2004). Recombinant human erythropoietin and its protein variants are used clinically for the correction of anemia secondary to CKD (Jelkmann 2008). Administration of an erythropoiesis-stimulating agent (ESA) restores the appropriate stimulatory signal to erythroid progenitor cells located within the bone marrow, thereby treating the anemia, reducing the incidence of secondary sequelae, and notably reducing the requirement for blood transfusions.

Peginesatide (formerly known as Hematide™) is a synthetic, PEGylated, investigational, peptide-based ESA that has an amino acid sequence unrelated to endogenous EPO (Fan et al. 2006) and is currently being developed in the United States for the treatment of anemia due to CKD in patients on dialysis. Unlike currently marketed ESAs, peginesatide is a peptide rather than a protein. In addition, it is linked to a branched two 20 kDa polyethylene glycol (PEG) moiety to prolong systemic circulation and reduce enzymatic proteolysis, which permits less frequent dosing than the majority of currently approved ESAs. PEGylation also has the theoretical advantage of reducing immunogenicity by sterically shielding the peptide from the immune system (Katre 1990).

To enhance the interpretation of efficacy and toxicology findings and support clinical development, studies in the rat were designed to define the absorption, distribution, metabolism, and excretion (ADME) characteristics, including clearance; characterize tissue distribution and metabolism; and delineate the major route(s) and rate of excretion of peginesatide following a single dose of either unlabeled or [^14^C] -labeled drug administered by IV injection. In addition, the affinity of peginesatide for melanin was evaluated by comparing tissue distribution in albino and pigmented rats.

## Materials and methods

### Chemicals

**Figure 1:**
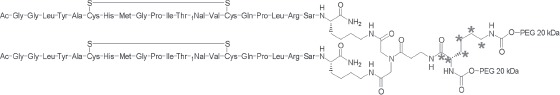
Chemical structure and sites of radiolabel for [^14^C]-peginesatide.

Peginesatide is a synthetic, peptidic ESA site-specifically conjugated to polyethylene glycol (Fan et al. 2006). Unlabeled and radiolabeled peginesatide was used in the studies. Radiolabeled [^14^C]-peginesatide, with specific activity ranging from 5.5-6.2 uCi/mg and a radiochemical purity of 89.6-91.4%, was synthesized by GE Healthcare (Buckinghamshire, UK). The [^14^C] radio-label was evenly distributed across the six carbons on the lysine linker between the peptide dimer and one of the two 20 kDa PEG chains (Figure 1). As a result of the radio synthetic procedure for the manufacture of the radiolabeled test article, the [^14^C]-peginesatide used in the studies described contained approximately 10% of peptide dimer conjugated to a single 20 kDa PEG designated mono-PEG. Due to analytical challenges associated with PEGylated compounds, the material was not able to be efficiently separated using preparative chromatography.

### Animals

Animal care for the studies conducted in the US was in compliance with Guide for the Care and Use of Laboratory Animals (NIH Publication 1996) and protocols were approved by the testing facility's Institutional Animal Care and Use Committee (IACUC). Similarly, the studies conducted in Japan were approved by the testing facility's Experimental Animal Ethical Committee.

Male Sprague-Dawley (albino) and Long Evans (pigmented) rats were approximately 8-11 weeks of age at the time of dosing and weighed between 240 and 300 g. Rats were housed with free access to standard chow and water unless fastingwas required for study-specific procedures. Animals were acclimated for at least a week prior to study initiation.

### Pharmacokinetics (absorption, distribution, metabolism, excretion) and mass balance: analytical methods

#### Enzyme linked immunosorbent assay

Plasma samples were analyzed for peginesatide concentration using a competition enzyme linked immunosorbent assay (ELISA) as previously described (Fan et al. 2006). The capture anti-peginesatide antibody was a high affinity polyclonal antibody preparation directed against the peptidic part of the molecule so the assay detects mono- and di-PEG-conjugated peptide as well as free peptide. The lower limit of quantitation was 40 ng-mL^1^, the intra assay precision (% coefficient of variation) was 5-10% and the intra assay accuracy (% bias) was 1.9-5.7%.

#### Radioactivity measurement

Radioactivity in diluted formulations, plasma, urine, and expired air (CO_2_-absorbent solution) was quantitated by mixingwith liquid scintillator Hionic-Fluor (PerkinElmer Life and Analytical Sciences) followed by liquid scintillation counting (LSC). Solid samples were homogenized (10 or 20% w/v in distilled water) before combustion in a sample oxidizer (A030701; PerkinElmer Life and Analytical Sciences) with Carbo-Sorb E (PerkinElmer Life and Analytical Sciences) as a carbon dioxide absorbent and Permafluor E^+^ (PerkinElmer Life and Analytical Sciences) as a liquid scintillator. Total captured radioactivity was subsequently measured via LSC. The recovery of radioactivity in the combusted samples was 96.9% or greater.

#### Quantitative whole body autoradiography

Tissue distribution of radioactivity was assessed by quantitative whole-body autoradiography (QWBA) in addition to determination of radioactivity tissue levels by LSC. At various time points following the administration of the desired QWBA target dose of 100 uCi/kg [^14^C]-peginesatide, equating to a 17mg/kg dose of peginesatide, the rats were anesthetized with isoflurane and blood (−3-4 mL) was collected via cardiac puncture. Anesthetized rats were sacrificed by immersion in a hexane dryicebath. Carcasseswere embedded in 2% carboxymethylceilulose over solid CO_2_ to ensure the block was retained frozen, and then stored at −20°C until sectioning. In addition, ^14^C radioactivity standards were embedded with each carcass to provide a context for quantification and for quality control purposes.

Samples were sectioned onto adhesive tape (Nakagawa NA-70 MAG, Japan) at five levels of interest in the sagittal plane, with all major tissues, organs, and biological fluids represented. Sections 40 urn thick were obtained using a Leica CM 3600 cryo-microtome at approximately −18°C and then dried in the cryo-microtome chamber. For each animal, a representative section from each level of interest was mounted on a thin cardboard support, placed on an aluminum support, and wrapped with mylar film. Fuji MS screens were exposed to the mounted sections along with a [^14^C]-autoradiographic standard, which allowed for subsequent calibration of the image analysis software. Exposed screens were scanned using the Molecular Dynamics Storm 820. To quantitate radioactivity in tissues, a calibration standard curve was constructed using American Radiolabeled Chemicals, Inc ARC 146 standards. The autoradiographic image data for the calibration standards and specified tissues, organs, and fluids were quantified using Imaging Research Inc. AIS software. Radioactivity concentrations in the sampled tissues were interpolated from each standard curve as nanocuries per gram. These concentration values were converted to microgram equivalents per gram of tissue on the basis of test material specific activity.

#### In vivo *metabolite profiling*

The metabolite profiling in the biological samples from rats dosed with [^14^C]-peginesatide was performed by high-performance liquid chromatography (HPLC). The radiolabeled materials in the samples of plasma and aqueous homoge-nates of tissues and feces were extracted with five volumes of methanol containing 0.1% of trifluoroacetic acid (TFA), mixed and centrifuged at 1500g at 4°C for l0 min to obtain supernatant. A portion of the supernatant was counted to estimate the extraction recovery of [^14^C]-peginesatide and its related compounds in the biological samples. The rest of the supernatant was evaporated to dryness under a nitrogen gas stream. The residue was re-dissolved in a small volume of equal parts mobile phase [MP(A)] and B [MP(B)] for chromatographic analysis. The HPLC system (Simadzu, Kyoto, Japan) consisted of a LC-lOADvp pump, a CTO-lOACvp column oven, an SPD-lOAvp UV detector, and an SCL-lOAvp system controller. HPLC separation was achieved at 40°C with a Zorbax 300SB-C8 column (150mm×2.1mm i.d., 5 μm, Agilent Technologies Japan, Ltd. Kyoto, Japan). A mixture of 0.1% TFA aqueous solution and 0.1% TFA in methanol solution were used for MP(A) and MP(B), respectively. The flow rate was 0.5mL/min, and the peaks were monitored by a UV absorption detector at 215 run. The time for the gradient elution was as follows: the concentration of MP(B) was increased from 50 to 80% and from 80 to 83% over a period of 2-10min and 10-20min, respectively. The concentration of MP(B) was continuously increased to 90% by 20.1 min and was held at 90% for lOmin, and then cycledbackto the initial condition (50%). The effluentwas collected into 0.5mL size fractions, followed by counting of the radioactivity (LSC). Under these conditions unchanged peginesatide and mono-PEG eluted at approximately 22 and 10 min, respectively.

### Pharmacokinetics

The PK of peginesatide was assessed in male Sprague-Dawley rats following the administration of unlabeled and radiolabeled test article. The linearity of the PK profile was evaluated in rats (*n* = 3/group) administered a single IV dose of peginesatide at 0.1, 0.5, or 5 mg·kg^−1^. Blood samples were collected predose and 0.25,1, 6, and 24h, and 2,3, 5, 7,10,14,17, 21, 24, and 28 days following dosing. Samples were analyzed by ELISA.

Following a single IV administration of 5 mg·kg^−1^ [^14^C] -peginesatide to rats (*n* = 3), blood samples were collected at 0.25, 1, 6, and 24h and 2, 3, 5, 7, 10, and 14 days after dosing. The plasma samples were analyzed for total radioactivity using LSC.

#### Pharmacokinetic analyses

Noncompartmental PK parameters for peginesatide and metabolites were generated using WinNonlin ver. 4.0, Pharsight Corporation (Mountain View, CA). The maximum plasma concentration (C_max_) and their times of occurrence (*t*_max_) were obtained directly from the mean plasma concentration vs. time profiles. The areas under the plasma concentration-time curves (AUC_0-1_) were estimated by the linear trapezoidal rule - by adding all the area portions under the curve from time zero to the time of the last observed plasma concentration (*t*_n_). Elimination rate constants *(kz)* were estimated by fitting a linear regression of log mean concentration against time data in the terminal exponential phase (at least 3 data points). Apparent terminal half-life *(t_1/2_)* was calculated as In2/λz. The AUC_0-inf_ was calculated as the sum of AUC_0-t_ and C_obs/λz_, where C_obs_, was the actual concentration at the time of the last quantifiable concentration. The plasma clearance (CL) for peginesatide was estimated as the ratio of dose/ AUC_0-inf_. The mean residence time (MRT) was calculated as the ratio of AUMC_0-inf_/AUC_0-inf_, where AUMC_0-inf_ was the area under the first moment curve from the time zero to infinity. The volume of distribution at steady state (*V_ss_)* was estimated as CL x MRT.

### Distribution

#### Concentration of radioactivity determined by LSC following combustion

Male Sprague-Dawley (albino) rats (*n* = 21; three per time point) were administered a single IV dose of 5 mg·kg^−1^ [^14^C]-peginesatide (27.5 μCi/kg) and tissue concentrations of radioactivity determined at 0.25, 6, and 24 h, and 7,14,30 and 60 days following dosing. Similarly, male Long Evans (pigmented) rats (*n* = 21; three per time point) were administered a single IV dose of 5 mg-kg^−1^ [^14^C]-peginesatide and plasma, skin, and eye concentrations of radioactivity were measured at the various time points through 60 days after dosing. At each time point, blood was collected, the animals sacrificed, and tissues excised. Tissues collected from the Sprague-Dawley rats included blood, plasma, adrenals, bone marrow, brain, eyes, fat, femur, Harderian glands, heart, hypophysis, intestine, kidneys, lungs, liver, pancreas, skeletal muscle, skin, spinal cord, spleen, stomach, submaxillary glands, testes, thymus, thyroid gland, and urinary bladder. Plasma, eyes, non-pigmented skin, and pigmented skin were collected from Long Evans rats. All tissue samples were measured for radioactivity by LSC following combustion. The liver and intestine were first homogenized in distilled water (20% w/v) prior to analysis. The concentration of radioactivity in the blood, plasma, and tissues was calculated and expressed as ug equiv·g^-1^ or mLbased on the LSC counts and the specific activity of the dosing solution.

##### Concentration of radioactivity determined by quantitative whole body autoradiography

Male Sprague-Dawley rats received a single IV (*n*=13; one per time point) dose of approximately 17 mg-kg"^1 ^[^14^C]-peginesatide. Following IV administration, tissue radioactivity concentrations were determined by QWBA at 0.25, 0.5, 1, 2, 4, 8, and 24h and 3 (2 rats), 5, 7, 14, and 28 days after dosing. Blood was collected at each time point and radioactivity concentrations were determined in blood and plasma by oxidation/LSC analysis in addition to QWBA determination of blood radioactivity concentrations. The concentration of the radiolabel in the blood, plasma, and tissues was calculated and expressed as ug equiv·g^-1^ or mL.

#### Mass balance study

The radioactivity excretion profile in urine, feces, and expired air of male Sprague-Dawley rats (*n* = 3) was characterized following a single IV administration of 5 mg-kg"^1 ^[^14^C] -peginesatide. Urine and feces were collected at 24-h intervals up to 14 days after dosing and expired air was collected up to 24 h after dosing. The levels of radioactivity in urine and expired air were measured by LSC and in feces by LSC following combustion. After the final collection of excreta at 2 weeks, the animals were sacrificed and the carcasses were dissolved in potassium hydroxide/distilled water/ethanol (3:3:7, w/v/v), and examined for residual radioactivity by LSC.

#### *In vivo* metabolite profiling *Plasma*

The plasma metabolic profile of [^14^C]-peginesatide was determined by the HPLC analysis described above and measurement of radioactivity using LSC. Samples were obtained from the animals (*n* = 3) utilized in the single dose PK study. Individual animal samples were pooled for each time point. The moieties detected were designated as peginesatide or mono-PEG (peginesatide with one PEG chain), with the remaining radioactivity designated as “Other” The classification of Other is anticipated to encompass smaller PEGylated-peptide and peptide/ amino acid components. The ug equiv/mL was calculated from the radioactive concentration and specific activity of the dosed formulation. The proportion of radioactivity for metabolite vs. total radioactivity in the plasma samples was estimated and metabolite concentrations calculated and expressed in ug equiv/mL. AUC values for the metabolite(s) were estimated based on plasma radioactivity vs. time data.

#### Urine and feces

The urine and fecal samples were obtained from the animals (*n* = 3) dosed in the mass balance study. Urine and fecal samples were collected every 24h and were combined into 2 aliquots (0-168 and 168-336h) for metabolite analysis. The urine and fecal samples were subjected to analysis by HPLC-fraction collection. Urine sample radioactivity was measured by LSC and fecal homogenate samples were measured by LSC following combustion.

#### Tissues

A single dose of [^14^C]-peginesatide at 5 mg·kg^−1^ was administered to male Sprague-Dawley rats (*n* = 9) by IV injection. At 14 days after dosing, animals were euthanized and samples of plasma, liver, spleen, kidneys, testes, and bone marrow were collected. Plasma and tissue extract samples were analyzed for levels of total radioactivity, peginesatide, and potential metabolites using LSC. Tissue homogenates (prepared in saline at a concentration of 20% [w/v] for the liver, kidneys and testes and 10% [w/v] for the spleen and bone marrow) were analyzed for radioactivity by LSC following combustion. The concentration of radioactivity in the blood, plasma, and tissues was calculated and expressed as ug equiv·g^-1^ or mL based on the LSC counts and the specific activity of the dosing solution.

## Results

### Pharmacokinetics

**Figure 2:**
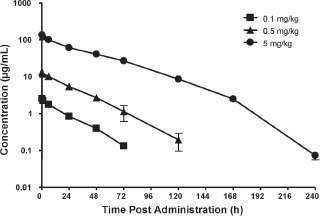
The plasma concentration-time profiles of peginesatide in male Sprague-Dawley rats after a single IV administration of peginesatide at a dose of 0.1, 0.5, or 5 mg·kg^−1^. Plasma concentration of peginesatide was determined by ELISA. Each data point represents the mean ± SD for three rats.

Pharmacokinetics were evaluated in rats following a single IV dose of peginesatide at 0.1, 0.5, and 5 mg·kg^−1^ (Figure 2, Table 1). Plasma concentrations of peginesatide were quantifiable for 3 days at the low dose of 0.1 mg·kg^−1^ and up to 7 days at the 5 mg·kg^−1^ dose. The maximum observed plasma concentration (C_max_) values increased in a dose-proportional manner and the increase in the area under the concentration-time curve (AUC) was generally greater than dose proportional across the entire dose range. Elimination half-life *(t_1/2_)* tended to increase with dose.

**Table 1:** Mean pharmacokinetic parameters of peginesatide in the plasma of male Sprague–Dawley rats after IV administration.

Dose (mg·kg^−1^)	C_max_ (μg/mL)	t_½_ (h)	AUC_0–t_ (μg·h/mL)	CL (mL/h/kg)	V_ss_ (mL/kg)
0.1	2.56 (0.162)	17.5 (0.3)	60.3 (4.71)	1.65 (0.13)	39.6 (3.03)
0.5	13.5 (1.40)	19.7 (2.6)	388 (48.8)	1.29 (0.18)	39.5 (2.06)
5	137 (6.19)	31.6 (3.4)	5405 (327)	0.92 (0.06)	39.6 (2.49)

Each value represents the mean for three animals, values in parentheses represent SD.

The *C*_max_ after intravenous administration represents the value at 0.25 h after administration.

Clearance (0.92-1.65 mL/h-kg) and volume of distribution at steady state (V_ss_) values were very low (39 mL/ kg). The clearance values were less than 1% of the rat glomerular filtration rate and the V_ss_ approximated the plasma volume (Lee and Blaufox 1985), suggesting limited distribution outside of the vascular compartment (Davies and Morris 1993; Lee and Blaufox 1985).

Pharmacokinetics of drug-derived radioactivity was also assessed following a single 5 mg-kg^-1^ IV [^14^C]-peginesatide administration. The PK parameters (C_max_ of 150 ug equiv·mL^−1^ and AUC_0-14 days_ of 6198 μg equiv h·mL^−1^) derived for total radioactivity were consistent with the PK parameters calculated based on plasma levels of peginesatide measured by ELISA. The ELISA quantifies most of the drug-related material that is present (mono- and di-PEGylated and free peptide); hence, the levels were similar to those of the radioactivity-based analysis.

### Distribution

#### Radiolabeled tissue distribution study

Radiolabel was detected in all tissues evaluated beginning 0.25 h after dosing following IV administration of [^14^C]-peginesatide to Sprague-Dawley (i.e. albino) rats, but the finding was considered to primarily reflect radio-label within the vasculature of the various tissues. The concentrations of radioactivity for selected tissues and time points are provided in Table 2. Distribution between the vascular compartment and the tissues was very slow as demonstrated by tissue to plasma ratios that were < 1 through at least 24 h after dosing, and >1 in most tissues thereafter. Consistent with the *V_ss_* data (minimal distribution), there was minimal, but observable, distribution into interstitial fluid in tissues, with slower distribution from tissues to plasma than vice versa. The C_max_ for several tissues, including blood, plasma, bone marrow, lung, and kidneys, was observed at 0.25 h after dosing.

**Table 2:** Concentrations of the radioactivity in the tissues of albino rats after a single IV administration of 5 mg·kg^−1^ [^14^C]-peginesatide.

	Concentration (μg equiv·g^−1^ or mL) at time after dose administration						
Tissues/organs	0.25 h	6 h	24 h	7 Days	14 Days	30 Days	60 Days
Blood	80.9 ± 1.90	60.9 ± 4.36	39.7 ± 2.51	3.87 ± 0.291	1.03 ± 0.229	0.466 ± 0.054	0.170 ± 0.036
Plasma	131 ± 3.86	99.6 ± 2.94	64.1 ± 5.12	7.80 ± 0.541	2.00 ± 0.605	0.780 ± 0.099	0.232 ± 0.042
Brain	1.18 ± 0.030	0.939 ± 0.015	0.646 ± 0.050	0.200 ± 0.021	0.102 ± 0.014	0.080 ± 0.010	0.057 ± 0.006
Spinal cord	0.909 ± 0.289	0.662 ± 0.070	0.442 ± 0.090	0.129 ± 0.067	0.069 ± 0.030	0.033 ± 0.016	0.036 ± 0.018
Pituitary	13.2 ± 1.08	13.9 ± 2.71	13.7 ± 2.02	8.31 ± 0.594	6.25 ± 1.17	7.24 ± 0.292	4.32 ± 0.289
Eyes	0.346 ± 0.029	0.779 ± 0.168	0.806 ± 0.063	0.885 ± 0.111	0.765 ± 0.022	0.511 ± 0.147	0.299 ± 0.076
Harderian glands	1.15 ± 0.357	1.91 ± 0.183	2.55 ± 0.105	4.41 ± 0.100	5.21 ± 0.433	3.88 ± 0.907	2.75 ± 0.281
Submandibular lymph nodes	2.57 ± 0.038	4.25 ± 0.140	5.72 ± 0.303	10.6 ± 0.553	10.9 ± 1.32	7.86 ± 1.19	8.74 ± 1.22
Thyroid gland	8.25 ± 1.42	10.1 ± 1.79	10.2 ± 1.10	17.0 ± 5.26	13.2 ± 3.52	13.9 ± 3.33	8.03 ± 1.45
Thymus	0.669 ± 0.115	1.02 ± 0.040	1.90 ± 0.201	3.39 ± 0.560	1.81 ± 0.249	1.14 ± 0.138	1.15 ± 0.155
Heart	6.33 ± 0.512	7.71 ± 1.29	6.28 ± 0.256	4.01 ± 0.344	2.73 ± 0.254	1.57 ± 0.244	1.14 ± 0.205
Lungs	8.46 ± 0.824	8.37 ± 1.27	7.22 ± 0.464	4.26 ± 0.285	3.38 ± 0.703	1.78 ± 0.237	1.05 ± 0.238
Liver	7.03 ± 0.613	7.51 ± 0.438	9.26 ± 0.516	12.5 ± 0.727	8.53 ± 0.754	3.45 ± 0.808	1.17 ± 0.199
Spleen	4.62 ± 0.185	5.51 ± 0.776	10.2 ± 1.04	11.5 ± 0.204	23.6 ± 2.82	17.7 ± 4.96	3.40 ± 0.480
Pancreas	2.56 ± 0.036	3.83 ± 0.582	5.14 ± 0.582	9.74 ± 0.231	9.10 ± 0.629	6.67 ± 1.70	5.92 ± 0.436
Adrenals	8.45 ± 0.517	7.83 ± 1.38	10.0 ± 1.15	10.2 ± 1.31	9.15 ± 2.39	7.90 ± 1.57	5.32 ± 0.996
Kidneys	11.2 ± 1.48	10.6 ± 1.16	8.75 ± 1.47	5.68 ± 0.386	4.49 ± 1.02	3.03 ± 0.429	2.11 ± 0.574
Testes	1.14 ± 0.083	4.01 ± 0.254	4.15 ± 0.605	4.98 ± 0.222	5.19 ± 0.448	4.33 ± 1.00	5.10 ± 2.17
Skeletal muscle	0.569 ± 0.100	0.447 ± 0.037	0.584 ± 0.044	0.563 ± 0.119	0.379 ± 0.108	0.152 ± 0.012	0.085 ± 0.021
Skin	0.376 ± 0.103	1.41 ± 0.175	3.29 ± 0.308	3.06 ± 0.055	1.70 ± 0.109	0.889 ± 0.228	0.394 ± 0.080
Fat	0.861 ± 0.096	0.923 ± 0.308	1.65 ± 0.392	2.11 ± 0.693	1.14 ± 0.475	0.582 ± 0.070	0.456 ± 0.135
Bone (femur)	1.95 ± 0.128	1.53 ± 0.555	1.16 ± 0.177	0.788 ± 0.374	0.388 ± 0.054	0.278 ± 0.057	0.046 ± 0.001
Bone marrow	9.66 ± 0.367	8.66 ± 2.79	8.50 ± 1.23	8.87 ± 0.641	6.33 ± 0.683	4.12 ± 1.37	0.361 ± 0.142
Urinary bladder	2.40 ± 1.32	3.53 ± 0.234	6.18 ± 0.260	4.52 ± 1.01	2.30 ± 0.326	1.77 ± 0.889	0.967 ± 0.188
Stomach	1.32 ± 0.257	2.69 ± 0.013	3.01 ± 0.168	2.36 ± 0.262	2.00 ± 0.118	1.28 ± 0.106	0.590 ± 0.242
Intestine	1.47 ± 0.158	3.07 ± 0.233	3.10 ± 0.144	3.05 ± 0.259	1.78 ± 0.297	1.40 ± 0.183	0.624 ± 0.033

Each value is the mean ± SD for three animals.

Following C_max_, radioactivity levels generally declined slowly over the course of the study with radioactivity still measurable in most tissues at 60 days after dosing. Plasma and blood concentrations decreased by approximately 2-fold at 24h and approximately 17- to 21-fold 7 days after dosing from C_max_ values of 131 and 80.9 ug equiv/mL, respectively. The average blood:plasma ratio across all time points was 0.6 indicating peginesatide was largely confined to the plasma fraction. The radioactivity level in the bone marrow, however, was relatively constant from 0.25 h through 7 days (∼9 ug equiv-g"^1^) and then gradually decreased over the remainder of the study (60 days).

The radioactivity in the other tissues, particularly glandular tissues, increased over time with C_max_ values observed from 6h to 14 days following administration of radiolabeled peginesatide to albino rats. The tissue with the highest C_max_, apart from blood, was the spleen, with maximum radioactivity concentrations of 23.6 ug equiv·g^−1^ at 14 days after dosing. Other tissues with high C_max_ values ranging from approximately 6-17 μg equiv·g^−1^ included the bone marrow, thyroid, pituitary, heart, sub-mandibular lymph nodes, lungs, liver, kidneys, bladder, adrenals, and pancreas. The C_max_ values for the remaining tissues were less than approximately 5 ug equiv·g^−1^. Radioactivity was still detectable in all tissues at 60 days after dosing with values ranging from 0.036 to 8.74 ug equiv·g^−1^.

**Figure 3:**
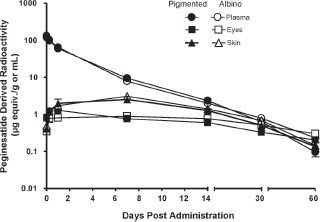
Log plasma concentration of the peginesatide-derived radioactivity in the plasma, eyes and skin of male albino (Sprague-Dawley) and pigmented (Long Evans) rats as a function of time after IV administration of 5 mg·kg^−1^ [^14^C]-peginesatide. Each value represents the mean ± SD for three animals.

The kinetics of an IV dose of radiolabeled peginesatide in plasma, skin and eyes of pigmented and non-pigmented rats were comparable, indicating that neither peginesatide nor the other radioactive components associated with melanin. A comparison of the values obtained in the albino and pigmented rats is outlined in Figure 3.

#### Quantitative whole body autoradiography study

**Figure 4:**
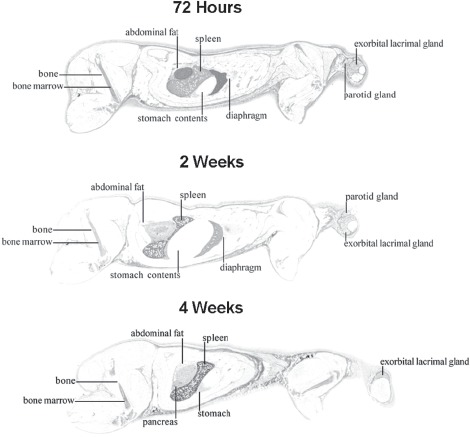
Whole-body sections and subsequent autoradiographs of male Sprague-Dawley rats 72h, 2 and 4 weeks following IV administration of 17 mg·kg^−1^ [^14^C]-peginesatide. Dark areas represent high radioactive concentrations.

Consistent with PK of unlabeled peginesatide, a plasma half-life of 27.8 h was observed in QWBA rats. Radioactivity appeared to distribute slowly between plasma and tissue, as in the tissue distribution study, and increased over time in some tissues. Localization of radiolabled material in tissues was observed in several tissues, such as spleen. The QWBA images obtained 72 h, 2 weeks and 4 weeks after dosing are presented in Figure 4. The QWBA data, in conjunction with the PK data, indicate that although radioactivity was detected throughout the majority of tissues except brain tissue protected by the blood brain barrier, the radioactivity appeared to be primarily confined to the vasculature, with slow distribution of the radiolabeled compound between the vascular compartment and tissues. Blood and plasma radioactivity remained fairly constant through 4h after dosing then gradually decreased. Elimination of drug-derived radioactivity from blood and plasma, following peak concentrations at 0.25 h after dosing, occurred in parallel with essentially constant blood to plasma ratios (0.52-0.58) observed through 672 h after dosing. Consistent with the whole tissue distribution data, a blood:plasma ratio of <0.55 indicated that peginesatide was largely confined to the plasma fraction.

The tissues that exhibited high concentrations at one or more time points included blood; the bone marrow (target tissue for pharmacologic effect); highly vascular-ized and lymphatic tissues such as the lung, liver, myocardium, spleen, and choroid plexus; sites of extramedullary hematopoiesis (EMH) in the rat, such as the spleen, adrenal gland, liver, kidney, and lymph nodes (Greaves and Faccini 1992; Elmore 2006); and organs/tissues associated with excretion of peginesatide including the kidney, bladder, and urine.

**Figure 5:**
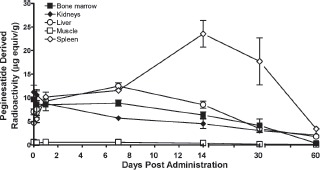
Biolocalization of peginesatide to hematopoietic and EMH sites compared to muscle following a single IV administration of 17 mg·kg^−1^ [^14^C]-peginesatide. Each data point represents the mean ± SD for three rats.

The findings of biolocalization to hematopoietic sites, including EMH, were consistent for both the traditional tissue distribution study (tissues are excised and radioactivity measured), as depicted in Figure 5, and QWBA studies. Levels of radioactivity remained relatively constant in the bone marrow throughout the study duration reaching an apparent C_max_ of 51.3 ug equiv·g^−1^ at 0.5 h after dosing. By 3 days after dosing, radioactivity levels in the bone marrow had only slightly decreased to 32.2 ug equiv/g and were 9.2 ug equiv·g^−1^ at 28 days. There was differential distribution of radioactivity within organs known to exhibit inducible EMH in rats, including the spleen (red pulp), adrenal gland, and kidney (medulla). Maximum differential distribution in the splenic red pulp was approximately 5-fold higher than in the white pulp. High concentrations of radioactivity were measured in the liver, an organ that exhibits EMH following pegi-nesatide administration to rats (Woodburn et al. 2008; Woodburn et al. 2009). There was no direct evidence of biliary excretion, but the recovery of radioactivity in the feces suggests that the radiolabel was secreted into the intestine via the bile or across the mucosa (Caliceti and Veronese 2003).

### Mass balance

**Figure 6:**
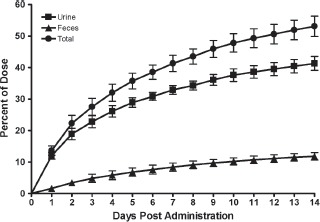
Cumulative recovery of peginesatide-related radioactivity in urine and feces of male rats following IV administration of 5 mg·kg^−1^ [^14^C]-peginesatide. At 2 weeks after dosing, the remaining approximately 50% of the radioactivity not excreted was detected in the carcass. Each value represents the mean ± SD for three animals.

The major route of excretion was in the urine with lesser amounts present in the feces (Figure 6). The excretion ratios of the radioactivity in the expired air were less than 0.1% of the dosed radioactivity in all animals by 24 h after dosing. Consequently, sampling of the expired air was terminated at 24 h. At 2 weeks after dosing, approximately 41 and 12% of the dosed radioactivity had been recovered in urine and feces respectively, with a further 50% in the carcass indicating that elimination was incomplete. The maximal excretion of radioactivity occurred during the initial 3 days after dosing, characterized by urinary and fecal recovery of 23 and 5% of the total radioactive dose, respectively. For the remainder of the study, the total daily excretion of radioactivity ranged from approximately 1 to 5%.

### Metabolism

Peginesatide, mono-PEG and remaining radioactivity, based on AUC_0_ *_u_*_da__s_ values, represented approximately 91, 4, and 6%, respectively, of the total radioactive exposure in plasma (Table 3). Remaining radioactivity (Other) is anticipated to be smaller PEGylated-peptide and pep-tide/amino acid components. Consistent with reduced PEGylation, the mono-PEG possesses a shorter *t_½α_*. half-life of 12.9 h compared to 29.1 h for peginesatide. At early plasma sampling time points (through 24 h), the relative radioactivity concentration of the mono-PEG appeared to primarily reflect the amount present in the dosing solution suggesting faster clearance of the dosing impurity compared to peginesatide. However, an extended *t* of 74 h was observed (Table 3), relative to a *t_1/ia_* half-life of 12.9 h, suggesting elimination of mono-PEG from tissues over time.

**Table 3:** Pharmacokinetic parameters of peginesatide and associated radioactivity in plasma after single IV administration of [^14^C]-peginesatide at a dose of 5 mg·kg−1 to rats.

Compound	C_max_ (μg/mL)	T_max_ (h)	t_½α_^a^ (h)	t_½β_^b^ (h)	AUC_0–14 days_ (μg·h/mL)
Total radioactivity	150	0.25	28.3	109	6335 (100)
Peginesatide	132	0.25	29.1	110	5743 (90.7)
Mono-PEG^c^	11.9	0.25	12.9	74	231 (3.6)
Other	–	–	–	–	360 (5.7)

a 0.25–72 h (peginesatide, total radioactivity), 0.25–48 h (mono-PEG).

b 168–336 h (peginesatide, total radioactivity), 72–240 h (mono-PEG).

Figures in parentheses represent proportions of the radioactivity (%).

c Dosing solution contained approximately 10% mono-PEG. n = 3.

- = not estimated.

Peginesatide, mono-PEG and Other radioactivity comprised 66.6, 32.7 and 0.7% of the urinary radioactivity, respectively, in urine samples collected over 336 h (Table 4). The percent dose excretion of mono-PEG in urine increased from 28.3 at 0-168h to 50% at 168-336h. A large proportion of the mono-PEG associated radioactivity present in the urine up to 168h after dosing likely represents elimination of mono-PEG present as an impurity in the dosing formulation. Mono-PEG associated radioactivity in the urine was also derived from mono-PEG elimination from tissues over time. In the feces collected up to 336 h after dosing, peginesatide, mono-PEG and Other comprised 16.1, 0.0 and 83.9% of the fecal radioactivity, respectively.

**Table 4:** Percent dose excretion of [^14^C]-peginesatide and associated radioactivity in rat urine and feces after single IV administration of 5 mg·kg^−1^.

	Urine			Feces		
Compound	0–168 h	168–336 h	0–336 h	0–168 h	168–336 h	0–336 h
Total Radioactivity	32.9 (100)	8.4 (100)	41.3 (100)	8.4 (100)	3.4 (100)	11.8 (100)
Peginesatide	23.3 (70.8)	4.2 (50)	27.5 (66.6)	1.9 (22.6)	LOQ (0)	1.9 (16.1)
Mono-PEG	9.3 (28.3)	4.2 (50)	13.5 (32.7)	LOQ (0)	LOQ (0)	LOQ (0)
Other	0.3 (0.9)	0 (0)	0.3 (0.7)	6.5 (77.4)	3.4 (100)	9.9 (83.9)

Values in parenthesis represent percent radioactivity for a given tissue/matrix at the specified time interval. *n* = 3.

Dosing solution contained approximately 10% mono-PEG.

LOQ, below the lower limit of quantitation.

The predominant moiety at 14 days in the plasma was parent molecule, representing approximately 80% of the total plasma radioactivity with mono-PEG and Other accounting for 5 and 15.4%, respectively (Table 5). Approximately 54-66% of the total radiolabel concentrations in the liver, spleen, kidney, testes and bone marrow represented peginesatide. Mono-PEG accounted for approximately 32-46% of the total recovered tissue radioactivity. Other accounted for approximately 15% of the total plasma radioactivity and 0-6% of the total tissue radioactivity.

**Table 5:** Day 14 concentrations of peginesatide and associated radioactive compounds in rat plasma and tissues after a single IV administration of 5 mg/kg [^14^C]-peginesatide.

	Concentration (μg equiv·g^−1^ or mL)					
Compound	Plasma	Liver	Spleen	Kidneys	Testes	Bone marrow
Total radioactivity	2.44 (100)	8.91 (100)	23.4 (100)	4.88 (100)	4.05 (100)	5.53 (100)
Peginesatide	1.94 (79.6)	5.63 (63.2)	14.3 (61.2)	2.64 (54.1)	2.68 (66.0)	3.22 (58.2)
Mono-PEG*	0.122 (5.0)	3.11 (34.9)	7.59 (32.5)	2.24 (45.9)	1.31 (32.2)	2.31 (41.8)
Other	0.375 (15.4)	0.162 (1.9)	1.49 (6.3)	0.00 (0.0)	0.069 (1.8)	0.00 (0.0)

Values in parenthesis represent percent radioactivity for a given tissue/matrix. *n* = 3.

## Discussion

Erythropoietin is deficient in patients with CKD-associated anemia. Administration of ESAs, such as peginesatide, leads to activation of the appropriate signaling at the EPO receptor (Fan et al. 2006). Peginesatide produces dose-dependent increases in reticulocytes and hemoglobin levels following a single IV administration in rats and humans (Fan et al. 2006; Stead et al. 2006). The principal findings in the single and repeat dose toxicity studies are primarily attributed to the pharmacological effects of peginesatide and secondary sequelae when an ESA is administered to an initially normocythemic animal. The principal peginesatide-related findings in the toxicology studies conducted in rats for up to 6 months were associated with the primary pharmacology of peginesatide as an ESA (Woodburn et al. 2009). Administration of peginesatide to initially normocythemic animals results in exaggerated pharmacology characterized by pronounced, sustained hemoconcentration and secondary clinical pathology, gross, and microscopic changes.

The data from the current study indicate that the dose-dependent pharmacological responses to peginesatide are associated with dose-dependent increases in systemic exposure. A dose-proportional increase in C_max_ and greater than dose-proportional increase in AUC were observed across the dose range of 0.1-5 mg·kg^−1^ following IV administration to rats. Peginesatide elimination was slow and biphasic. The biphasic response was characterized by a relatively rapid elimination from the vascular compartment at the early time points, followed by a prolonged terminal phase. The concentration of radioactivity in plasma was higher than that in whole blood, with blood:plasma ratios of approximately 0.6 consistent with radioactivity being confined to the plasma (non-cellular) fraction and evidence of minimal, to no, red blood cell association. The slow elimination translated into sustained plasma and blood levels of peginesatide. The PK profiles, including elimination kinetics in blood and in plasma, were similar when based on total radioactivity compared to ELISA analysis consistent with the ELISA recognizing the pep tide portion of the molecule.

The nonlinear PK of peginesatide is suggestive of a saturable elimination process. Multiple clearance mechanisms may be involved with peginesatide as clearance involves glomerular filtration; a presumably non-saturable elimination. Renal glomerular filtration is consistent with the excretion data and the notable decrease in clearance of −57% for 5/6 nephrectomized vs. normal rats (Fan et al. 2006). The nonlinear kinetics of erythropoiesis stimulating proteins has been attributed to saturable clearance mechanisms involving EPO (Kato et al. 1997) and non-EPO receptor-mediated pathways (Agoram et al. 2009). Agoram et al. suggest that non-EPO receptor mediated pathways, which may involve clearance in the interstitium or lymphatic system, contribute to clearance and play a significant role in prolonged clearance, including that of PEGylated erythropoietins (Agoram et al. 2009).

Peginesatide is generally confined to the vascular compartment with the volume of distribution approximating the plasma volume (Davies and Morris 1993), which is consistent with high concentrations of radio-label being detected in highly vascularized tissues throughout the course of the distribution studies. There is some (slow) distribution between plasma and tissues, as evidenced by the shift in tissue/plasma ratios from <1 to >1 over time (suggesting slower distribution out of than into tissue).

Consistent with the erythropoietic properties of peginesatide, localization was observed in the bone marrow, the principal erythropoietic site in adult mammals. Bone marrow levels were sustained throughout the 28-day evaluation period (9.1 μg·g^-1^ tissue). For the radiolabel distribution and QWBA studies, the bone marrow to bone ratios ranged from approximately 11-25.

High concentrations of radioactivity were also associated with EMH sites consistent with putative EPO receptor location (Juul et al. 1998), which in the rat include spleen, adrenal gland, liver, lymph nodes, and kidney (Greaves and Faccini 1992; Elmore 2006). EMH, which appropriately occurs in the rat following increased demand or stimulation, is not a normal physiologic or pathophysiologic process that occurs in humans following birth. The QWBA analysis retains the architectural integrity of the organs and tissues and, in conjunction with high spatial resolution, demonstrates distribution details (e.g. biolocalization) of the radiolabel that is not apparent with LSC assessment. Specifically, radioactivity was distributed differentially in the renal medulla, the adrenal capsule and red splenic pulp. As noted, high concentrations of radioactivity were measured in the liver. Supporting a pharmacological response to peginesatide, functional activity was characterized by the histologic demonstration of EMH in both the spleen and liver following peginesatide administration in rats (Woodburn et al. 2008; Woodburn et al. 2009).

Lymphatic tissues also exhibited among the highest concentrations of radioactivity at one or more time points following administration of radiolabeled peginesatide to the rat. The relatively high levels of radioactivity in the lymphatic tissues possibly reflect the uptake or removal of the macromolecule by lymphoid cells. Following administration, peginesatide may be transported to the draining lymph nodes (including the spleen) and may undergo potential processing and/or degradation of the peginesatide molecule (Porter et al. 1991).

Renal excretion is a major elimination route, with approximately 41% of the peginesatide-derived radioactivity recovered in the urine. The latter is consistent with a previous study in which peginesatide exhibited a twofold lower clearance in a rat chronic renal insufficiency model compared to that in a rat with normal renal function (Fan et al. 2006). Fecal elimination was observed (−12% of the radioactive dose), but to a lesser extent than renal excretion. High concentrations of radioactivity were also measured in the liver. In addition, negligible excretion (<0.1%) of the radiolabel was detected in the expired air, demonstrating stability of the radiolabel.

The *in vivo* metabolic pathway for proteins/peptides (Webster et al. 2007; Garibotto et al. 2010) as well as PEGylated protein therapeutics (Webster et al. 2007; Caliceti and Veronese 2003; Hamidi et al. 2006), is well characterized and understood. Regardless of species (including humans), proteins, peptides and PEGylated moieties undergo enzymatic proteolysis (hydrolysis) to yield smaller peptides or amino acids and/or are excreted via the kidneys or are incorporated into the amino acid pool for utilization in the subsequent biosynthesis of proteins (Garibotto et al. 2010). For CKD patients, excretion of amino acid constituents does also occur via hemodial-ysis (Garibotto et al. 2010). PEGylation sterically hinders enzymatic proteolysis, but ultimately, the conjugated protein/peptide undergoes proteolysis.

Peginesatide, mono-PEG and Other, defined as smaller PEGylated peptides and peptide/amino acid components, were identified in plasma, urine and tissues. The chemical mechanisms surrounding cleavage of a mono-PEG branch from a peptide linker has been elucidated in *in vitro* systems using mild base-catalyzed hydrolysis (Guiotto et al. 2004). Hydrolysis appeared to be responsible for the cleavage of one 20 kDa PEG moiety from a-interferon conjugated with a 40 kDa branched PEG similar to peginesatide. Following cleavage, the mono-PEGylated moiety was subsequently excreted in urine (Caliceti and Veronese 2003; Guiotto et al. 2004).

The level of mono-PEG present in the plasma at the early time points is considered to reflect the level generated as an impurity during the manufacture of the [^14^C]-labeled material and present in the dosing formulation, with mono-PEG levels at later time points reflecting elimination from tissues. Parent molecule and mono-PEG comprised 67 and 33%, respectively, of the radioactivity present in urine collected over 336 h after dosing. The increased proportion of total urine radioactivity associated with mono-PEG at later time points corresponded with tissue elimination of the molecule. A small proportion of the 12% of the dose recovered in feces was parent molecule with the majority reflecting smaller PEGylated-peptide, and peptide/amino acid components.

Of the remaining radioactive dose at 14 days, the majority of radiolabel in the spleen, liver, kidney, tes-tes and bone marrow was associated with the parent molecule (−54-66%) compared to either the mono-PEG (−32-46%) and smaller PEGylated peptide and peptide/amino acid components (−15%). The mono-PEG presence represents degradation of peginesatide in the tissues over time.

The totality of the metabolism data is consistent with *in vitro* studies conducted in liver and kidney micro-somes and S9 fractions from rats, monkeys, and human donors (Im et al. 2010). Peginesatide was the main component measured following incubation with microsomes from all species and no metabolites were observed, demonstrating metabolic stability *in vitro.*

At 14 days after dosing, approximately 50% of the radioactive dose remained in the body. Despite these retention levels, no direct drug toxicity has been observed with peginesatide administration in chronic rat toxicology studies in which rats were administered up to 20 mg/kg every 3 weeks for 6 months (Woodburn et al. 2009). The toxicities observed were consistent with pronounced, sustained polycythemic-related effects and are a function of exaggerated pharmacology following administration of an ESA to initially normocythemic animals. The erythro-poietic activity of mono-PEG has been demonstrated to be substantially less than the activity associated with the parent molecule (data not shown), which is attributed to decreased circulatory exposure of the mono-PEGylated molecule. The pathophysiological processes occurring with peginesatide and which are secondary to the hemo-concentration are not anticipated to occur in anemic CKD patients whose ESA dose levels are individually titrated to maintain Hgb levels below 11 g/dL, below physiological Hgb levels, and with a gradual increase over time.

## Conclusions

In summary, the studies and results described provide a comprehensive delineation of the peginesatide absorption, distribution, metabolism and excretion profile in the rat. The data demonstrate that administration of peginesatide results in sustained systemic exposure, which is consistent with the robust erythropoietic response observed in rats and humans given peginesatide (Fan et al. 2006; Stead et al. 2006). Elimination of peginesatide from the plasma exhibited a biphasic pattern typical of PEGylated compounds characterized by a relatively rapid initial phase and a prolonged terminal phase. The QWBA data, in conjunction with the PK data, indicate that although radioactivity was detected throughout the majority of tissues, the radioactivity appeared to be primarily confined to the vasculature with slow distribution of the radiolabel between the vascular compartment and tissues. The tissues that exhibited high concentrations at one or more time points included blood; highly vascularized and lymphatic tissues, sites of hematopoi-esis and EMH, and organs/tissues associated with excretion of peginesatide including the kidney and bladder. Peginesatide's pronounced persistence within bone marrow may contribute to the extended pharmacologic action. A predominant degradation event to occur *in vivo* is the loss of one PEG chain from the branched PEG moiety to generate mono-PEG. In addition, peginesatide and mono-PEG undergo hydrolysis to smaller PEGylated-peptide and peptide/amino acid components. Urinary excretion was a major elimination route with parent molecule representing the primary moiety excreted.
